# Impact of a Mental Health Consultation Program on Child Psychosocial Development over Two School Years

**DOI:** 10.3390/bs15111497

**Published:** 2025-11-04

**Authors:** Ruby Natale, Yue Pan, Yaray Agosto, Carolina Velasquez, Elana Mansoor, Rebecca Jane Bulotsky-Shearer, Sarah E. Messiah, Jason F. Jent

**Affiliations:** 1 Mailman Center for Child Development, University of Miami Miller School of Medicine, Miami, FL 33136, USA; yagosto@med.miami.edu (Y.A.); cxv308@miami.edu (C.V.); emansoor@med.miami.edu (E.M.); jjent@med.miami.edu (J.F.J.); 2 Department of Public Health Sciences, University of Miami Miller School of Medicine, Miami, FL 33136, USA; panyue@med.miami.edu; 3 Department of Psychology, University of Miami, Coral Gables, FL 33146, USA; rshearer@miami.edu; 4 Department of Epidemiology, Peter O’Donnell Jr. School of Public Health, University of Texas Southwestern Medical Center, Dallas, TX 75390, USA; sarah.messiah@utsouthwestern.edu; 5 Department of Pediatrics, Children’s Health System of Texas, University of Texas Southwestern Medical Center, Dallas, TX 75390, USA

**Keywords:** early childhood mental health consultation, childcare, prosocial skills, challenging behaviors, social-emotional development

## Abstract

High-quality early care and education (ECE) programs, characterized by safe environments, emotionally supportive communication, proactive behavior supports, and teacher self-care practices, play a pivotal role in healthy child development. Early Childhood Mental Health Consultation (ECMHC) is an evidence-based approach designed to strengthen these environments and support young children’s social–emotional outcomes. However, the long-term impacts of ECMHC models remain understudied. Grounded in ECMHC, the purpose of this study is to evaluate the effectiveness of Jump Start Plus COVID Support (JS+CS) in supporting child psychosocial outcomes (prosocial behaviors and reduced externalizing/internalizing behaviors) over two school years. In a cluster-randomized trial, 12 ECE centers received the 14-week JS+CS intervention, and 12 attention control centers received a 14-week obesity prevention program. Children were followed over two school years to determine long-term impacts on behavior, measured by the Devereux Early Childhood Assessment (DECA) and the Strengths and Difficulties Questionnaire (SDQ). Over two school years, significant time-by-group interactions emerged for primary child outcomes. The JS+CS group showed greater improvements in DECA Initiative and Self-Regulation (*p* = 0.01 and *p* = 0.02, respectively) compared to controls. JS+CS significantly enhanced child psychosocial functioning, supporting its potential as an effective model for a scalable mental health consultation in ECE settings.

## 1. Introduction

High-quality early care and education (ECE) programs, characterized by safe environments, emotionally supportive communication, proactive behavior supports, and teacher self-care practices, play a pivotal role in healthy child development (Natale et al., 2023b). Because up to 70% of a child’s waking hours are spent in ECE centers, these settings are vital for cultivating early social–emotional competencies that shape long-term well-being and academic success (Harding & Paulsell, 2018; Hartman et al., 2017). Furthermore, high-quality ECE programs have been shown to prevent behavior challenges in children from low-income areas (Gilliam et al., 2016). Many ECE programs, especially in under-resourced communities, struggle with significant challenges, with child behavioral problems among the most pressing (Hoffman & Kuvalanka, 2019). These concerns have intensified in the wake of the COVID-19 pandemic, as more children have been identified as having behavioral issues (Mullins et al., 2025).

Recent research indicates this increase reflects both a greater number of children exhibiting challenging behaviors and a higher frequency and intensity of these behaviors across multiple domains. Studies have documented elevated rates of both externalizing (e.g., aggression, defiance, and tantrums) and internalizing behaviors (e.g., anxiety, withdrawal, and emotional dysregulation) compared to pre-pandemic levels (Jing et al., 2024; Panchal et al., 2021). A meta-analysis of more than 38,000 preschoolers found the prevalence of emotional and behavioral problems rose to 24.3%, which was nearly a threefold increase from pre-pandemic rates of 6.9–14.7% (Jing et al., 2024). Importantly, these findings indicate that more children now present with behavioral and emotional needs warranting additional support within early childhood education settings.

The increased prevalence of behavior concerns places additional strain on ECE programs, particularly in low-resource communities, which must meet increasingly complex behavioral and social-emotional needs. Research on teacher stress indicates that 87% of teachers reported feeling overwhelmed by difficulties in maintaining a high-quality learning environment while addressing challenging behaviors (Natale et al., 2023b). Therefore, the purpose of this study was to test the effectiveness of an Early Childhood Mental Health Consultation (ECMHC) program aimed at improving child psychosocial functioning over time.

ECMHC is an evidence-based mental health consultation provided to childcare centers to reduce teacher stress and address children’s challenging behaviors (Gilliam et al., 2016; Natale et al., 2022b, 2025). ECMHC is a multi-level intervention (Duran et al., 2010; Raver et al., 2008) that has demonstrated positive impacts on both teacher and child outcomes across multiple implementation trials throughout the United States (Gilliam et al., 2016; Natale et al., 2022b, 2025). At the center level, ECMHC provides directors with strategies to develop center-wide policies related to behavioral supports (Duran et al., 2010). At the teacher level, ECMHC helps teachers develop strategies to overcome teaching challenges, strengthen their job-related self-efficacy, reduce teaching-related stress (Gu & Day, 2007), and improve classroom practices (Duran et al., 2010). At the child level, the consultation model provides teachers with skills to increase children’s prosocial skills and executive functioning (Bleecker et al., 2005; Downer et al., 2012; Duran et al., 2010; Gilliam, 2007; Perry et al., 2008).

Jump Start + COVID Support (JS+CS) is an example of an ECMHC developed in Miami-Dade County during the COVID-19 pandemic. The program is designed to im-prove child psychosocial functioning by promoting self-care, communication, and improving behavioral support strategies at the child, teacher, and center levels. Initial evaluation of the JS+CS program demonstrated significant immediate benefits for both children and educators following the consultation intervention. Children whose teachers participated in JS+CS showed enhanced prosocial skills in the period directly after pro-gram completion (Natale et al., 2025). Concurrently, JS+CS teachers experienced meaningful improvements across multiple professional competency domains, including significantly enhanced implementation of classroom safety practices and evidence-based approaches to managing challenging child behaviors compared to baseline measures (Natale et al., 2024). While these immediate JS+CS post-intervention results are encouraging and demonstrate the program’s short-term efficacy, questions remain regarding whether JS+CS improvements are sustained over extended periods.

Beyond immediate positive outcomes, multiple research trials have demonstrated that ECMHC models produce sustained beneficial effects for both teachers and children in childcare centers following intensive consultation periods. ECMHC follow up research demonstrated higher classroom quality, stronger teacher competence and coping skills, and lower staff turnover 6–8 months after teachers complete consultation services. ECMHC research has also documented long-term reductions in teacher-child conflict, decreased use of exclusionary discipline practices, and reduced racial disparities in disciplinary actions maintained at up to 9 to 12 months (Drake-Croft et al., 2025). Reductions in children’s aggressive behavior and suspensions have also been documented nine months following initial consultation (Upshur et al., 2009). These findings suggest the ECMHC model can promote enduring improvements in child behavior, classroom environment, and educator well-being. However, no study to date has tracked outcomes over two school years. Therefore, this is a critical gap in the literature and rigorous longitudinal studies are needed. Until such data is available, claims about the long-term effects of ECMHC remain promising but not supported with evidence. Therefore, the current study examined the long-term effects of an ECMHC consultation model, JS+CS on children’s behavior and prosocial skills across two school years.

### The Current Study

As a part of a larger cluster randomized controlled trial, we examined the extent that JS+CS positively impacted child outcomes over two school years (Natale et al., 2023a). Research Question 1: Do children whose teachers were randomized to the JS+CS condition differ in prosocial skills two years post-intervention from children whose teachers participated in the active control condition? Specifically, we hypothesized that teachers of children randomized to JS+CS will view children as exhibiting significantly higher levels of prosocial skills (i.e., attachment/relationships, initiative, self-regulation) over two school years relative to children assigned to an active control. Research Question 2: Do children whose teachers were randomized to the JS+CS condition differ in problem behavior two years post-intervention from children whose teachers participated in the active control condition? We hypothesized that teachers of children randomized to JS+CS would rate children as exhibiting significantly fewer total problem behaviors and fewer externalizing and internalizing behaviors over two school years relative to children assigned to an active control. Research Question 3: Do children whose teachers were randomized to the JS+CS condition demonstrate clinically significant differences as compared to Healthy Caregivers—Healthy Children (HC2) over two school years? We hypothesized that children in the JS+CS intervention would demonstrate greater clinically significant improvements in problem behaviors and prosocial skills over two school years compared to children in an active control group. High-quality childcare centers are essential settings for promoting lifelong mental health, social-emotional development, and school readiness, particularly for children in under-resourced communities. Findings from this research may inform sustainable, scalable public health strategies aimed at improving early childhood mental health, reducing disparities, and supporting the well-being of educators and children nationwide.

## 2. Materials and Methods

### 2.1. Sample

**Figure 1:**
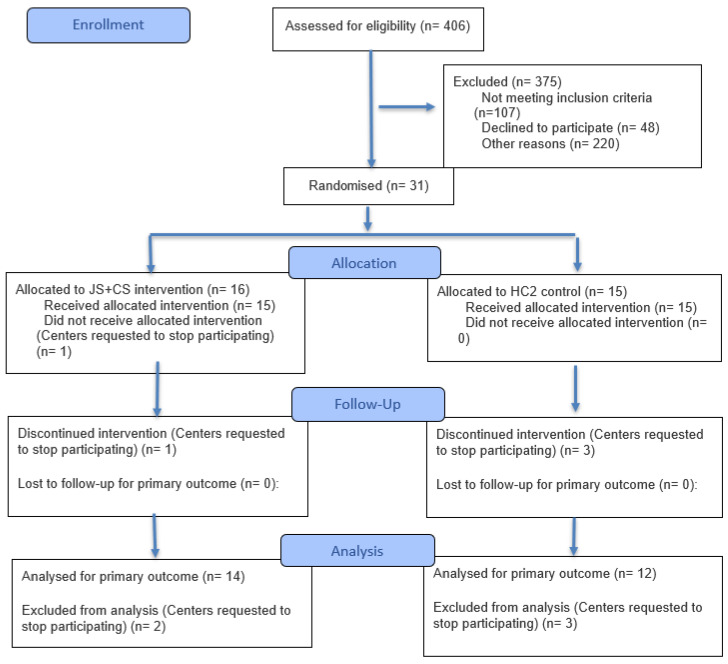
CONSORT Diagram.

This study was conducted in 30 ECE centers in South Florida, United States. Centers were eligible to participate if they met the following criteria: (1) had ≥50 children (≥30 of whom are 18 month-3 years old); (2) located in the low-income census tract, with at least 50% of families receiving childcare subsidy; (3) served at least 60% Hispanic or 60% Non-Hispanic Black families; (4) had directors and teachers willing to participate. Children/families were able to participate if they were in a classroom where the teacher was enrolled in the study; and (5) had not previously participated in an early childhood mental health consultation program. A total of 671 children participated in this study. See Table 1 for child baseline demographics. This study was approved by the University of Miami’s Institutional Re-view Board (IRB) and is currently registered with ClinicalTrials.gov (NCT05445518). All teachers and parents/guardians of the child participants signed informed consent prior to participating. See Figure 1. CONSORT diagram for enrollment, allocation, and analysis total numbers.

### 2.2. Measures

#### 2.2.1. Demographics

At baseline, parents/guardians were asked to complete a 33-item intake questionnaire designed to collect sociodemographic information about themselves and their child. Child-level variables included age, gender, race, ethnicity, English language proficiency, primary language, and health insurance coverage. Teachers completed both an individual intake form and a classroom demographics questionnaire, which captured information on their professional background, classroom composition, and reported suspension and expulsion practices.

#### 2.2.2. Child Prosocial Skill Measures

The teacher-reported Devereux Early Childhood Assessment (DECA) was administered at all four timepoints to assess children’s prosocial skills. These included the DECA for Infants and Toddlers (DECA-I/T; Mackrain et al., 2007) and the DECA for Preschoolers, Second Edition (DECA-P2; LeBuffe & Naglieri, 2012). Both instruments are validated, reliable, standardized, and norm-referenced teacher-report measures of protective factors that promote resilience in children between the ages of 1 month and 5 years (LeBuffe & Naglieri, 1999; Naglieri et al., 2013). There are 36 items on the DECA-I/T and 38 items on the DECA-P2 that are rated on a five-point scale from “never” (0) to “very frequently” (4). The measures yield three different subscales: Initiative (i.e., ability to use independent thoughts and actions to meet needs), Self-Regulation (i.e., ability to express emotions and express behaviors in healthy ways), and Attachment/Relationships (i.e., mutual, strong, long-lasting relationship between child and significant adults), as well as a Total Protective Factors (TPF) score. Each DECA item begins with the prompt “During the past 4 weeks, how often did the toddler/child...” followed by behaviors such as “calm her-self/himself down” and “play well with others.” Additionally, the DECA measures have adequate internal consistency in an English and Spanish speaking, low-income, diverse sample (Crane et al., 2011). T scores based on the national standardization sample for each scale were utilized for continuous variable analyses. Further, T scores were binary coded clinical concern levels (0 = Within normal limits; T score of 41 or higher; 1 = Clinical concern; T score of 40 or lower). In this study, the internal consistency for the DECA total protective factors scale at T1 was α = 0.975. The DECA was selected to assess child outcomes because it is a standardized measure consistently used across multiple iterations of the Jump Start program, allowing for longitudinal comparison across models and cohorts. In addition, the DECA has been widely used in the IECMHC literature to evaluate social-emotional outcomes and program impacts (Carlson & Voris, 2017; Bulotsky-Shearer et al., 2013).

#### 2.2.3. Child Externalizing and Internalizing Behaviors Measures

Similar to the DECA, the teacher-report version of the Strengths and Difficulties Questionnaire (SDQ) was used to evaluate children’s behavioral problems at all four time points. The SDQ is a 25-item behavioral screening tool developed for children and adolescents aged 2 to 17, with separate versions for the 2–4 and 4–17 age groups. It has also demonstrated promising reliability when used with younger children aged 12 to 24 months, with stronger reliability for externalizing than internalizing subscales (Patel et al., 2021). Items are rated on a three-point Likert scale ranging from “not true” (0), to “somewhat true” (1), and “certainly true” (2). Each item describes a specific child behavior, such as “Many worries or often seems worried,” “Easily distracted, concentration wanders,” and “Often offers to help others (parents, teachers, other children).” The Externalizing, Internalizing, and Total Problems scales were utilized for the current study. Based on SDQ published scoring categories, children’s scores for total problems, externalizing problems, and internalizing problems were categorized as being within the normal range (coded as 0) or categorized as borderline, high, or very high range (coded as 1) based on the scoring for the version completed. The SDQ has established validity and reliability (Goodman, 1997). In this study, the internal consistency for the SDQ total problems scale at T1 was α = 0.860. The SDQ was selected to assess child outcomes because it is a standardized measure consistently used in the ECMHC literature to evaluate social-emotional outcomes and program impacts (Silver et al., 2023; Ride et al., 2025). While item content is slightly different for 3 of 10 items on the Externalizing scale, all other items for Externalizing and Internalizing scales were the same. In addition, each scale had the same range of raw scores (0 to 20) across both SDQ versions.

#### 2.2.4. Teacher Consultations

A consultation was defined as a scheduled hybrid session (in-person and/or virtual) between a mental health consultant (MHC) or research assistant (RA) and a childcare center staff member that included the delivery or discussion of intervention content (e.g., JS+CS pillars infographics or HC2 lesson plans). Consultation dosage was calculated as the total number of sessions each teacher received across the two study years as documented on weekly consultation logs. For both arms, a minimum of 14 consultation notes were expected during Year 1 to reflect the 14-week intensive intervention period, and at least four additional consultations were anticipated in Year 2 to capture the quarterly booster sessions.

### 2.3. Procedures

The study was a randomized controlled trial in which 30 childcare centers were provided either the Jump Start program or HC2 (Natale et al., 2023a). Centers were block randomized by region of Miami-Dade County (north, central and south). The HC2 was chosen as an active control group given this is a more rigorous design as it controls for attention and contract verses using a standard control.

The procedures were consistent across groups in which trained MHCs and graduate-level RAs collected data at four timepoints, each approximately six months apart: Time 1 (T1) occurred at baseline, at the beginning of the first school year; Time 2 (T2) occurred post-intervention, at the end of the first school year; Time 3 (T3) occurred at the beginning of the second school year; and Time 4 (T4) occurred at the end of the second school year. MHCs and RAs were from corresponding cultural backgrounds and have experience working in their assigned area of the county. Center directors were recruited and consented first, followed by teachers, and then families. A “Meet and Greet” occurred weekly for the first 2 weeks for teachers and then 2 weeks later for parents, during which the study was explained by our program’s MHCs. Participants giving verbal agreement were contacted by the RA based on their preference for email or phone to obtain consent and collect measures during lunch or naptime (for teachers) and during drop off our pickup times (for parents) with an option for oral administration. All data was entered into REDCap, a secure, web-based application de-signed exclusively to support data capture for research studies (Harris et al., 2009).

#### 2.3.1. Jump Start Plus COVID Support (JS+CS) Description

The JS+CS program was specifically designed to address early childhood mental health needs during the COVID-19 pandemic (Natale et al., 2023a). Grounded in Bronfenbrenner’s Bioecological Systems Theory, the Georgetown Model recognizes that child development occurs within interconnected environmental systems encompassing family, educational, and broader sociocultural contexts (Bronfenbrenner, 1979, 1994). This theoretical framework informs the model’s multi-tiered intervention approach similar to the Georgetown model, which addresses systemic factors at the center, teacher, and child levels rather than focusing exclusively on individual child difficulties.

The JS+CS program maintains this ecological perspective, recognizing that challenging behaviors often stem from environmental factors such as ineffective communication patterns among staff, administrative challenges at the center level, and elevated teacher turnover resulting from occupational stress. Central to the JS+CS model is the emphasis on enhancing teacher competencies and instructional practices as the primary mechanism for improving child outcomes. The program’s core objective is to strengthen educators’ capacity to effectively respond to challenging behaviors while simultaneously fostering children’s social-emotional development. This teacher-focused approach recognizes educators as key agents of change within the child’s immediate learning environment.

JS+CS integrates Caring for Our Children national standards (American Academy of Pediatrics et al., 2019), CDC COVID-19 guidance (National Center for Immunization and Respiratory Diseases, 2021), and evidence-based strategies to promote children’s social competence (Hemmeter et al., 2022). The intervention is structured around four pillars: (1) Safety, (2) Behavior Support, (3) Self-Care, and (4) Communication. During Year 1, MHCs delivered content to directors (program-level) and teachers (classroom-level) through weekly one-hour sessions over a 14-week period. Due to pandemic-related restrictions, consultations were provided through a hybrid model using both in-person and telepresence technology. Each center received a comprehensive toolkit that included an action plan outlining weekly goals and strategies, which served as a standardized implementation protocol for MHCs. A typical consultation began with goal setting based on the pillar for that week, followed by the introduction of pillar-specific infographics and short animated “how-to” videos from the program website illustrating resiliency-based coping strategies for achieving those goals. Applying a reflective consultative stance, MHCs then guided teachers in practicing these strategies while providing real-time feedback. The toolkit included a total of 24 infographics aligned with the four pillars, along with supplemental classroom materials such as handwashing timers, No Yell bells, and See My Feelings mirrors. Each infographic served as a guide through the consultative process, following a standardized format: (1) Reflect, (2) Inform, and (3) Practice. For example, a Behavior Support infographic on classroom rules prompted teachers to reflect on how expectations are communicated to children, emphasized the use of simple, positively phrased rules with visual cues to promote prosocial behavior, and encouraged teachers to co-create and model classroom rules with children while providing positive reinforcement. During Year 2, MHCs provided four quarterly booster sessions (one per pillar) to center staff to help sustain their skills in supporting center-wide implementation of program pillars and policy standards.

#### 2.3.2. Healthy Caregivers—Healthy Children Control Description

Centers randomized to the active control arm received an existing obesity prevention program, Healthy Caregivers—Healthy Children (HC2) (Natale et al., 2022a) by trained consultants. This program has improved obesity prevention practices within other centers (Messiah et al., 2017; Natale et al., 2014, 2017). A doctoral level staff member with expertise in child nutrition and exercise habits trained the consultants. The HC2 program focused on four pillars: physical activity, snack, beverage, and screen time. Each childcare center received materials for implementation, including detailed lesson plans for each pillar to be integrated within daily classroom activities. Each lesson plan outlined the objective, preparation steps, relevant props, activities, language/vocabulary, and culturally and linguistically appropriate service associations and enrichment questions (Natale et al., 2022a).

An example lesson plan was the “Introduction to My Body”. The objective of this lesson was to introduce children to the My Body poster and provide a basic overview of the major organs/body systems. To engage children, teachers were instructed to use a puppet (prop), Healthy Howie, as a classroom “guest”. Healthy Howie served as the lesson’s narrator and guide. Teachers began the lesson by introducing Healthy Howie with a scripted prompt, such as “This is Healthy Howie! He is going to help us learn how to be healthy and strong. Today he will show is the orans in our bodies and explain what they do to keep us healthy.” Using the My Body poster as a visual aid, teachers highlighted key organs/systems, such as the brain, bones, muscles, and other organs. Children were encouraged to share prior knowledge by responding to prompts (e.g., “Does anyone know the name of an organ or part inside the body?”). Teachers also displayed supplementary pictures of body systems to reinforce learning and provide visuals for discussion. The lesson combined interactive elements with visual supports to encourage engagement and comprehension among the young children.

During Year 1, the HC2 program was implemented across 14 weeks via hybrid (in-person and/or virtual) consultations lasting approximately one hour. During Year 2, implementation consisted of four booster consultations, also delivered in a hybrid approach lasting approximately one hour every other month. Each consultation focused on a condensed version of each pillar with key points and strategies.

### 2.4. Statistical Analysis Plan

Baseline characteristics were summarized using descriptive statistics stratified by treatment group (JS+ CS vs. HC2). For continuous variables, means and standard deviations were reported; for categorical variables, frequencies and percentages were calculated. Group differences at baseline were assessed using independent *t*-tests for continuous variables and chi-square tests for categorical variables, excluding observations with missing treatment assignment.

#### 2.4.1. Research Questions 1 and 2

To examine differences in longitudinal child outcomes between the two intervention groups (JS+ CS vs. HC2) over time, we conducted Generalized Estimating Equations (GEE) with repeated measures for each of the seven child outcomes: prosocial skills comprised DECA Attachment, Initiative, Self-Regulation, and Total Protective Factors; and child externalizing and internalizing behaviors comprised SDQ Externalizing Problems, Internalizing Problems, and Total Difficulties. All outcomes were modeled as continuous variables using an identity link function and assuming normally distributed errors. The working correlation matrix was specified as compound symmetry to account for with-in-subject correlations across time points. Each model included fixed effects for treatment group (JS+CS vs. HC2), time (T1 through T4), and the interaction between treatment group and time. We also included the following baseline covariates: child age at intake, primary language at intake, and total number of teacher consultations received across all timepoints, as well as the baseline value of the outcome variable. Site-level clustering was accounted for by nesting record ID within program name as the repeated subject. Estimated marginal means (least square means) and standard errors were computed by treatment group and time point to assess adjusted group differences. Type III Wald chi-square tests were used to assess the significance and relative effect size of the main effects (treatment and time) and the treatment-by-time interaction.

Because multiple related outcomes were tested, we additionally applied the Benjamini–Hochberg False Discovery Rate adjustment to control for potential Type I error inflation. Given the conceptual and statistical correlation among DECA and SDQ domains, this approach was preferred over more conservative family-wise error corrections (e.g., Bonferroni). False discovery rate-adjusted p-values were examined to confirm the robustness of significant findings.

#### 2.4.2. Research Question 3

To complement analyses when using the SDQ and DECA scales as continuous variable outcomes, we also examined change over time using dichotomized outcomes to examine changes in children’s who met the threshold for clinically low levels of prosocial behavior or high levels of problem behavior. Binary indicators were created based on established published clinical cutoffs for the DECA and the SDQ described in the measures section (0 = Within normal limits; 1 = Clinical concern). We modeled these dichotomous outcomes using GEE with a logit link function and binomial distribution, adjusting for the same baseline covariates, and using the same repeated subject structure as previously described in the continuous outcome models. Odds ratios (ORs) with 95% confidence intervals were estimated to quantify the magnitude of group differences in the proportion of children achieving clinically meaningful change. Type III chi-square tests were again used to assess the significance and effect size of treatment group, time, and their interaction in the binary outcome models. All analyses were conducted using SAS 9.4 (SAS Institute, Cary, NC, USA) and visualized using R (R Foundation for Statistical Computing, Vienna, Austria).

#### 2.4.3. Sensitivity Analysis for Baseline Imbalance

Because treatment groups differed significantly at baseline on several sociodemographic variables (e.g., age, gender, race/ethnicity, primary language), we conducted propensity score (PS) sensitivity analyses to assess the robustness of our findings to potential confounding due to baseline imbalance. Propensity scores were estimated using logistic regression with treatment group (JS+ CS vs. HC2) as the outcome and child baseline characteristics (age, gender, race, ethnicity, primary language) and total teacher consultations as predictors. Missing categories were retained using CLASS statements with the MISSING option to preserve sample size. Stabilized Inverse Probability of Treatment Weights (IPTW) were then calculated from the estimated propensity scores. To minimize the influence of extreme weights, we applied 1st/99th percentile trimming.

Weighted GEE models were re-estimated for each outcome using the same specifications as the primary analyses (continuous outcomes: identity link, normal distribution; binary outcomes: logit link, binomial distribution). Each model included baseline outcome, child age, and language covariates, with clustering by program. Standardized mean differences (SMDs) were calculated before and after weighting to evaluate covariate balance, with |SMD| < 0.10 considered acceptable.

#### 2.4.4. Sensitivity Analysis for Attrition Bias

Because attrition from T1 to T4 was substantial, we conducted an Inverse Probability of Censoring Weighting (IPCW) analysis to assess the robustness of findings to potential bias due to differential dropout. Logistic regression models were used to estimate each participant’s probability of remaining in the study through T4 based on baseline covariates, including treatment arm, child age, gender, race, ethnicity, primary language, and baseline DECA and SDQ scores. Stabilized censoring weights were calculated as the ratio of the marginal probability of completion to the predicted probability of completion, with weights trimmed at the 1st and 99th percentiles. These IPCW weights were then applied to the GEE models to adjust for potential bias from non-random attrition.

## 3. Results

### 3.1. Baseline Characteristics

Baseline characteristics by treatment group are presented in Table 1. The JS+CS group consisted of 304 children, while the HC2 group included 367 children. Children in the JS+ CS group were slightly older on average (mean = 3.59 years, SD = 1.18) than those in the HC2 group (mean = 3.34 years, SD = 1.15; *p* = 0.0068). A higher proportion of children in the HC2 group were male (55.2%) compared to the JS+ CS group (44.3%, *p* < 0.0001). There were significant group differences in race, ethnicity, and primary language (all *p* < 0.0001). Most children in both groups spoke either English or Spanish, with a larger proportion of Spanish-speaking children in the JS+ CS group (68.1%) compared to HC2 (51.7%). The JS+ CS group had more teacher consultations between T1 and T4 (mean = 18.71, SD = 5.97) compared to HC2 (mean = 11.96, SD = 4.32; *p* < 0.0001).

### 3.2. Child Outcomes over Time

**Figure 2:**
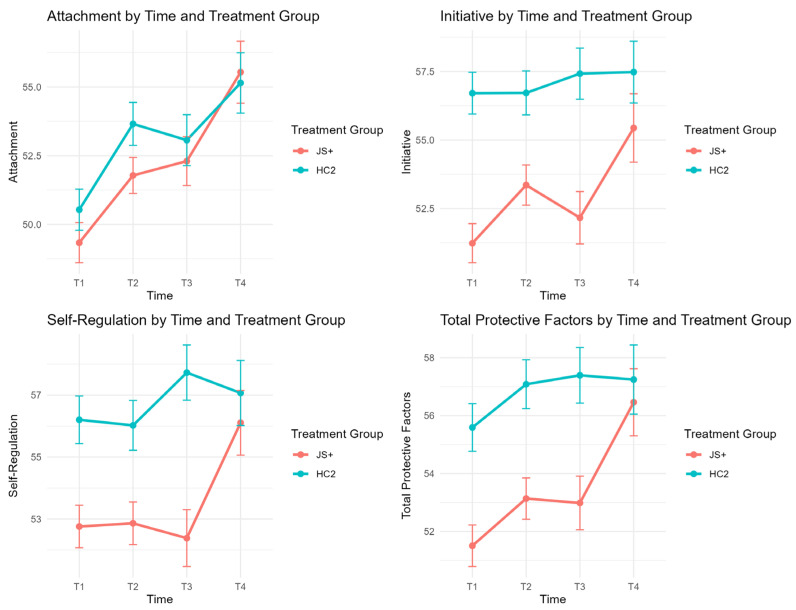
DECA outcomes over time by treatment group.

**Figure 3:**
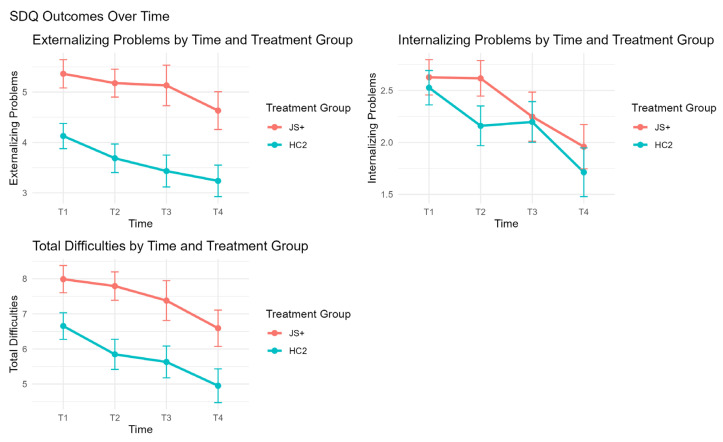
SDQ outcomes over time by treatment group.

Table 2 presents unadjusted means and standard errors for each outcome by group and time point and GEE model results controlling baseline values and covariates. Visual representations of these results are presented in Figure 2 and Figure 3.

Across all four time points, children in the HC2 group consistently demonstrated higher DECA scores than those in the JS+ group. After adjusting for baseline, covariates, and site clustering, for DECA Self-Regulation, the HC2 group showed significantly higher scores than JS+ with an adjusted mean difference of −2.21 (SE = 0.79, *p* = 0.0054, χ^2^ = 6.71). Similarly, DECA Initiative showed a significant treatment-by-time interaction (χ^2^ = 10.49, *p* = 0.0148) with group differences favoring HC2 at T4 (See Figure 2). GEE tests revealed a significant main effect of time for DECA Self-Regulation (*p* = 0.0054), with additional significant time-by-treatment interactions observed for DECA Initiative (*p* = 0.0148) and Self-Regulation (*p* = 0.0269), suggesting that these outcomes changed differentially over time by group (See Figure 2). That is, the rate of improvement was greater in the JS+CS group, demonstrating improved initiative and self-regulation, since the HC2 group maintained higher absolute mean scores for most of the time.

In terms of problem behavior outcomes, the JS+ group had statistically significantly higher SDQ Externalizing Problems (difference = 0.76, SE = 0.28, *p* = 0.0066, χ^2^ = 6.81) and significantly higher SDQ Total Difficulties (difference = 1.01, SE = 0.39, *p* = 0.0093, χ^2^ = 6.4), indicating more behavior problems in the JS+ group overall. A marginal difference was found for SDQ Internalizing Problems (difference = 0.31, SE = 0.18, *p* = 0.083, χ^2^ = 2.95) (See Figure 3). Again, these results indicate that the JS+ group started out with lower prosocial and higher problem behavior initially. No significant interactions were found for SDQ outcomes, indicating that trends over time were similar across groups (See Figure 3).

Although HC2 maintained higher DECA scores and lower SDQ scores over time, children in the JS+ group demonstrated clinically meaningful improvements over time in both DECA and SDQ outcomes. For DECA, the JS+ group showed steady increases from T1 to T4 in Total Protective Factors (from 51.50 to 56.46), Attachment (from 49.33 to 55.54), Initiative (from 51.24 to 55.44), and Self-Regulation (from 52.76 to 56.11). These gains narrowed the gap between JS+ and HC2 by the final time point. Similarly, SDQ scores among children in both treatment groups showed consistent improvement over time (Figure 2 and Figure 3). Additionally, the total number of teacher consultations was not associated with any of the outcomes and did not modify the treatment effect. Covariates such as child age and language at intake were included in the model but did not show consistent effects across outcomes.

### 3.3. Changes over Time in Clinical Levels of Behavior by Treatment Group

**Figure 4:**
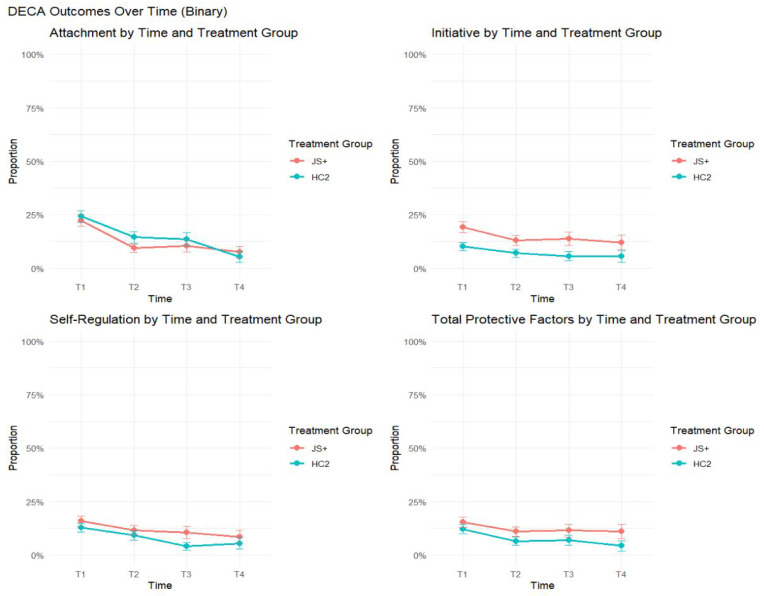
DECA binary outcomes over time by treatment group.

**Figure 5:**
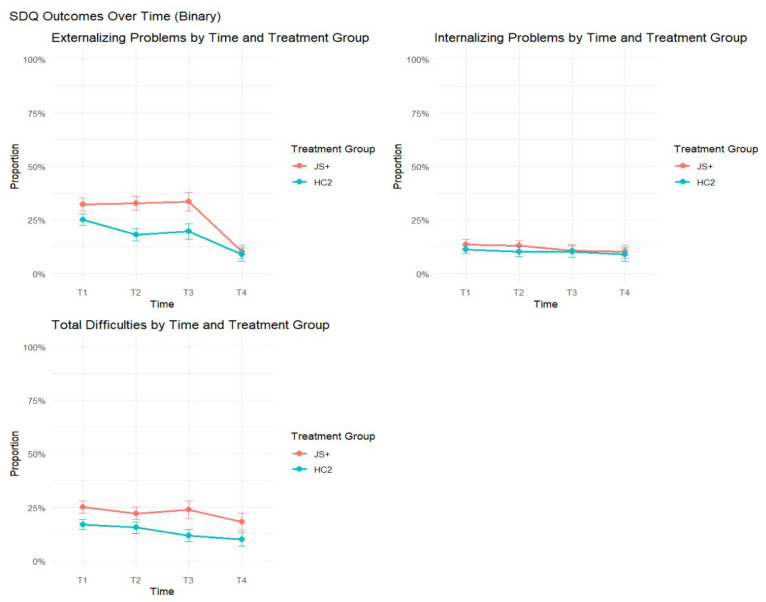
SDQ binary outcomes over time by treatment group.

When examining binary indicators of clinical levels of child prosocial skills and problem behaviors, there were no statistically significant differences between the JS+ and HC2 groups, either in overall treatment effects or treatment-by-time interactions (Table 3, Figure 4 and Figure 5). Both groups demonstrated gradual reductions over time in the proportion of children rated as experiencing clinically concerning levels, reflecting overall improvements in functioning. While the patterns were broadly consistent across binary SDQ and DECA outcomes, continuous outcome models showed that JS+ children exhibited greater gains over time in DECA Initiative and Self-Regulation compared to HC2 (Table 2, Figure 2), indicating some group differences in the trajectory of change even if not reflected in binary outcomes. Overall, the two groups showed similar patterns of change over time in clinical indicators, with both experiencing improvements, though some differential trends were observed in underlying continuous scores.

### 3.4. Results of the Sensitivity Analysis for Baseline Imbalance

To address baseline imbalances between treatment groups, we conducted IPTW analyses (Appendix A Table A1). Results were broadly consistent with the primary GEE findings.

### 3.5. Results of the Sensitivity Analysis for Attrition Bias

To assess potential bias due to participant attrition over time, we conducted IPCW analyses (Appendix A Table A2). Logistic regression models examining predictors of study completion at T4 showed that older children were significantly less likely to have follow-up data available (OR = 0.50, 95% CI [0.39, 0.64], *p* < 0.001). Baseline DECA Total Protective Factors and SDQ Total Problems were not significant predictors, though higher SDQ problem scores showed a marginal trend toward lower retention. No demographic or treatment group differences in attrition were observed.

## 4. Discussion

This study provides promising evidence for the long-term effectiveness of the JS+CS model in improving child prosocial skills within childcare centers. Over two school years, children in childcare centers that received JS+CS demonstrated significantly greater improvements in both Initiative and Self-Regulation, as measured by the DECA. These gains suggest that children in the JS+CS group became more capable of independently meeting their needs and managing their emotions and behaviors across settings, key indicators of developing social-emotional competence. These findings extend previous short-term outcomes, in which the JS+CS approach, which centers on reflective consultation and classroom support for teachers, can promote positive shifts in children’s prosocial skills (Natale et al., 2025). From a public health perspective, enhancing child prosocial skills in early childhood, particularly within under-resourced childcare settings, can serve as a protective factor against later academic failure, mental health challenges, and involvement with exclusionary discipline practices, underscoring the broader societal value of scalable, ECMHC models like JS+CS. Even after applying both IPTW to adjust for baseline imbalances and IPCW to account for attrition, the overall pattern and magnitude of effects remained consistent with the primary models. These findings suggest that the study results are robust to potential biases arising from both baseline confounding and differential dropout, supporting the internal validity of the observed treatment effects.

Although positive changes were demonstrated on the DECA for JS+CS compared to HC2, the SDQ did not show significant treatment-by-time interactions. This may partially be due to a primarily non-clinical sample, where all group means fell within normative ranges on both measures, which may have limited the magnitude of observable change due to potential ceiling effects. Further, despite cluster randomization, there was a higher percentage of children in the JS+CS group with clinical-level externalizing concerns on the SDQ at study entry (32.2% vs. 25.2% in HC2) which may have influenced the results. Conversely, the majority of the children in the HC2 group were not in the clinically significant range on the SDQ at baseline. Nevertheless, clinically meaningful improvements were observed within the JS+CS group. The proportion of children with externalizing behavior clinical concerns dropped substantially from 32.2% at T1 to just 10.2% at T4, representing a 68% relative reduction. This finding is notable, as early externalizing behaviors are among the strongest predictors of preschool suspension and expulsion (Gilliam et al., 2016), particularly in under-resourced settings. While between-group comparisons were not statistically significant, these within-group gains suggest that JS+CS may be effective in reducing risk for exclusionary practices in early childhood settings. Future studies with larger sample sizes and stratified randomization procedures are warranted to more precisely evaluate these effects and mitigate baseline imbalances.

An alternative explanation for the lack of significant differences over time between treatment groups in teacher-rated problem behaviors on the SDQ may lie in the measure’s scoring approach and developmental alignment with the target population. The SDQ uses raw scores, whereas the DECA provides norm-referenced T-scores, which are more sensitive to detecting change over time and facilitate standardized comparisons across children (LeBuffe & Naglieri, 1999). In addition, the researchers had to use two different SDQ versions, to cover the full range of children in our sample. This decision ensured that each child was assessed using items that were developmentally relevant and consistent with the SDQ’s administration guidelines. However, the use of multiple versions introduces the potential for minor measurement non-equivalence across time. Although the internalizing and externalizing subscales contain the same number of items, response scales, and total score ranges, differences in age-specific normative cutoffs could contribute to variance unrelated to true developmental or intervention effects. In our analyses, children’s clinical classifications were based on the version-specific cutoffs recommended for each form, which preserved developmental validity but may limit strict comparability across assessments. Future studies could employ harmonization techniques such as z-score standardization, item response theory linking, or multi-group confirmatory factor analysis to enhance longitudinal measurement consistency across SDQ versions. In contrast, the DECA provides developmentally tailored versions for ages 1.5–3 and 3–5, which more precisely align with our study population. The DECA’s emphasis on early childhood social-emotional competencies and behavioral risk makes it particularly suitable for assessing preschool-aged children and may account for greater sensitivity to intervention effects in this age group.

Beyond measurement considerations, it is also important to recognize that the HC2 program, though an obesity prevention program, may have influenced child behavior indirectly, through its emphasis on health-promoting classroom practices. In addition, HC2 may have reduced the observed effect size leading to an underestimation of the JS+CS actual impact. HC2 emphasizes improvements in classroom practices related to physical activity, nutrition, and screen time, all of which may be associated with child behavioral and emotional outcomes. For instance, structured physical activity in early childhood settings has been linked to reductions in hyperactivity and enhancements in attention span, directly targeting externalizing behaviors such as impulsivity and aggression (Becker et al., 2014). Future research should more explicitly examine how health-focused interventions like HC2 may influence child behavior and prosocial skill alongside health outcomes, particularly in settings where integrated approaches to health and behavior may offer dual benefits for young children. Further, future cluster randomized trials of JS+CS should consider employing alternative active control conditions that have demonstrated minimal influence on children’s behavioral or prosocial outcomes, such as injury prevention or creative storytelling programs.

Consistent with prior research on ECMHC, these results reinforce the critical role of early intervention strategies embedded within naturalistic learning environments (Futterer et al., 2024; Natale et al., 2023b). Similarly, a study on the efficacy of a home/preschool intervention for children with challenging behaviors found an increase in children’s social skills, and a reduction in behavior problems. However, that study was conducted with a different demographic, predominantly Caucasian children. Moreover, it was based on the Preschool First Step to Success model which is different from the current study’s ECMHC model (Feil et al., 2014). Another study based on the Pyramid Model conducted with a small sample of three teachers and three children also showed a reduction in challenging behaviors by the children and high acceptability from the teachers (Rakap & Balikci, 2024). However, the small sample size of that study warrants caution for generalizability. In contrast, the current study expands the field by demonstrating that the benefits of ECMHC-based interventions can be maintained over an extended period, an area previously understudied. Notably, the improvements observed in both DECA and SDQ clinical concern prevalence rates across treatment groups provide a dual lens on positive skill-building (e.g., prosocial behavior and self-regulation) and the reduction of risk (e.g., externalizing and internalizing symptoms), which are central goals of effective early childhood mental health support. 

Overall, the success of JS+CS in improving children’s prosocial skills may be partially attributed to its integration of mental health consultation with existing classroom practices, promoting teacher capacity to proactively support children’s emotional and behavioral needs. Literature shows that teacher practices are essential in supporting child development. (Duran et al., 2010; Yoshikawa et al., 2013) The findings align with the growing consensus that ECE programs are not only educational spaces but also essential public health settings (Harding & Paulsell, 2018; Hartman et al., 2017). By enhancing teacher practices, relational communication, and behavioral support structures, JS+CS fosters environments that buffer children against early developmental risk and promote resilience. 

### 4.1. Limitations

Despite these promising results, several limitations warrant consideration. The study was limited to 30 centers in one geographic region, which may affect generalizability. In addition, the use of two different SDQ versions for different age groups within the same longitudinal study is problematic and could introduce variance unrelated to developmental change or intervention. We chose to use the SDQ because it was a standard measure used in the field when evaluating IECMHC and that is within the age range of children in this study (Ride et al., 2025; Silver et al., 2023). However, future researchers should use caution when using this measure across age groups. In addition, the DECA is a standardized measure commonly used to evaluate outcomes in IECMHC (Carlson & Voris, 2017; Bulotsky-Shearer et al., 2013); however, because its norms are over 10 years old, future research should consider incorporating more recently normed measures. Additionally, while teacher practices were a key target of the intervention, this study focused on child outcomes; future analyses should examine the mediating role of teacher behavior in driving child-level change. A key limitation of the current study is the substantial attrition from T1 to T4, which reduced the overall sample size and may limit the generalizability of findings. However, to address this, we used GEE for all longitudinal analyses. GEE is a robust statistical approach that allows for the inclusion of all available data at each time point without requiring complete data across all waves, thereby minimizing bias due to random missingness (Zeger & Liang, 1986). Despite randomization at the center level, baseline differences in child sociodemographic characteristics may have introduced residual confounding. Although models adjusted for these factors and sensitivity analyses using IPTW and IPCW produced consistent results, statistical adjustment cannot fully eliminate bias due to baseline nonequivalence or informative dropout. Both methods assume that all relevant confounders and predictors of missingness were adequately measured, which may not hold in practice. Future studies with larger, more balanced samples and enhanced follow-up procedures are needed to further reduce bias and strengthen causal inference. Lastly, while the control group received an attention-matched intervention (obesity prevention), further studies should compare JS+CS to other mental health or behavioral interventions to refine our understanding of its relative effectiveness.

### 4.2. Implications

Longer-term ECMHC outcome data are critical for policy and funding decisions. Early childhood stakeholders require evidence of sustained return on investment to justify resource allocation for ECMHC programs. Without rigorous longitudinal data extending beyond one school year, support for ECMHC’s long-term effectiveness remains promising but inadequately substantiated for evidence-based practice standards (Center of Excellence for Infant and Early Childhood Mental Health Consultation, 2022; Zinsser et al., 2022). For example, Caring for our Children National Health and Safety Standards provides a national framework of best practice polices to enhance the quality of care in childcare centers. EMCHC strategies, such as those embedded in the JS+CS model, could inform and integrate with these national best practice standards. JS+CS practices could institutionalize reflective supervision, mental health promotion and family communication practices, ensuring that ECMHC principles are sustained beyond the individual consultation practices.

### 4.3. Public Health Impact

Access to quality childcare is an overlooked but critical social determinant of health (Iruka, 2020). It has the power to significantly benefit children through improved psychosocial support. Implementing a culturally and linguistically responsive program such as JS+CS can prepare childcare centers for dealing with long-term effects of COVID-19 and other public health crises through strengthening centers’ safety, self-care, communication, and behavior practices. Improving children’s prosocial skills can significantly help these disparate communities weather long-term effects.

## 5. Conclusions

This study adds to the growing body of evidence supporting ECMHC-based models as effective, scalable strategies for enhancing child mental health and development in early care settings. While our study findings are promising, future studies are required for further confirmation from more methodologically rigorous studies (e.g., stratified randomization approach to reduce risk of baseline differences between groups, more rigorous follow-up data collection strategies to reduce missing data). To our knowledge, our study is one of the first in the field to demonstrate that mental health consultation can have long-term impacts over two school years. Results suggest that JS+CS offers a sustainable pathway for strengthening ECE program quality through targeted teacher consultation and support, with measurable, lasting benefits for the children they serve.

## Figures and Tables

**Table 1:** Baseline Characteristics by Treatment Group.

Characteristic	JS+ (*N* = 304)	HC2 (*N* = 367)	*p*-Value
Age, years, M (SD)	3.59 (1.18)	3.34 (1.15)	0.0068
Gender, n/N (%)			<0.0001
Female	160/287 (55.7)	142/317 (44.8)	
Male	127/287 (44.3)	175/317 (55.2)	
Race, n/N (%)			<0.0001
White	206/278 (74.1)	198/307 (64.5)	
Non-Hispanic Black	48/278 (17.3)	87/307 (28.3)	
Native American	4/278 (1.4)	4/307 (1.3)	
Asian Pacific Islander	1/278 (0.4)	0/307 (0.0)	
Multiracial	14/278 (5.0)	9/307 (2.9)	
Other	5/278 (1.8)	9/307 (2.9)	
Ethnicity, n/N (%)			<0.0001
Hispanic	238/284 (83.8)	217/315 (68.9)	
Non-Hispanic White	11/284 (3.9)	16/315 (5.1)	
Non-Hispanic Black	24/284 (8.5)	60/315 (19.0)	
Haitian	7/284 (2.5)	18/315 (5.7)	
Other	4/284 (1.4)	4/315 (1.3)	
Primary Language, n/N (%)			<0.0001
English	90/285 (31.6)	151/317 (47.6)	
Spanish	194/285 (68.1)	164/317 (51.7)	
Creole	1/285 (0.4)	2/317 (0.6)	
Total Teacher Consults, M (SD)	18.71 (5.97)	11.96 (4.32)	<0.0001

Note: Sample sizes vary due to missing data.

**Table 2:** Child Outcomes by Treatment Group and Time (Continuous Outcome).

Outcome	JS+ T1 M (SE)	JS+ T2 M (SE)	JS+ T3 M (SE)	JS+ T4 M (SE)	HC2 T1 M (SE)	HC2 T2 M (SE)	HC2 T3 M (SE)	HC2 T4 M (SE)	JS+ vs. HC2 Diff (SE)	Chi Square Diff	Difference *p*-Value	Chi Square-Tx × Time	Tx × Time *p*-Value
DECA Attachment	49.33 (0.73)	51.78 (0.65)	52.30 (0.89)	55.54 (1.13)	50.53 (0.75)	53.66 (0.78)	53.06 (0.93)	55.14 (1.10)	−0.26 (0.88)	0.09	0.7664	3.44	0.3289
DECA Initiative	51.24 (0.71)	53.36 (0.73)	52.16 (0.96)	55.44 (1.25)	56.71 (0.76)	56.72 (0.80)	57.42 (0.93)	57.48 (1.13)	−1.18 (0.86)	1.77	0.171	10.49	0.0148
DECA Self-Regulation	52.76 (0.69)	52.86 (0.69)	52.38 (0.92)	56.11 (1.04)	56.20 (0.77)	56.02 (0.80)	57.73 (0.89)	57.07 (1.05)	−2.21 (0.79)	6.71	0.0054	9.19	0.0269
DECA Total Protective Factors	51.50 (0.72)	53.13 (0.71)	52.98 (0.93)	56.46 (1.16)	55.59 (0.82)	57.08 (0.84)	57.39 (0.96)	57.25 (1.20)	−1.01 (0.87)	1.26	0.2459	6.24	0.1006
SDQ Externalizing Problems	5.36 (0.28)	5.18 (0.28)	5.13 (0.40)	4.63 (0.37)	4.13 (0.25)	3.69 (0.28)	3.43 (0.32)	3.24 (0.31)	0.76 (0.28)	6.81	0.0066	0.60	0.8972
SDQ Internalizing Problems	2.63 (0.17)	2.62 (0.17)	2.25 (0.24)	1.96 (0.21)	2.53 (0.17)	2.16 (0.19)	2.20 (0.20)	1.71 (0.23)	0.31 (0.18)	2.95	0.083	0.47	0.9247
SDQ Total Problems	7.99 (0.39)	7.79 (0.40)	7.38 (0.57)	6.59 (0.52)	6.65 (0.38)	5.85 (0.43)	5.63 (0.45)	4.95 (0.48)	1.01 (0.39)	6.4	0.0093	0.41	0.9372

Note. JS+ = Jump Start + COVID Support; HC2 = Healthy Caregivers, Healthy Children (Active Control); DECA = Devereux Early Childhood Assessment; SDQ = Strengths and Difficulties Questionnaire. Values are estimated marginal means with standard errors in parentheses. Diff = group difference with standard error. Tx = treatment.

**Table 3:** Child Outcomes by Treatment Group and Time (Binary Outcome).

Outcome	JS+ T1 Concern/ n (%)	JS+ T2 Concern/ n (%)	JS+ T3 Concern/ n (%)	JS+ T4 Concern/ n (%)	HC2 T1 Concern/ n (%)	HC2 T2 Concern/n (%)	HC2 T3 Concern/n (%)	HC2 T4 Concern/ n (%)	OR (95% CI)	Chi Square Difference	Difference *p*-Value	Chi Square Tx × Time	Tx × Time *p*-Value
DECA Attachment	53/237 (22.4%)	20/214 (9.3%)	12/116 (10.3%)	7/93 (7.5%)	61/251 (24.3%)	26/180 (14.4%)	17/126 (13.5%)	4/76 (5.3%)	0.61 (0.26–1.45)	0.09	0.2645	2.77	0.4286
DECA Initiative	45/234 (19.2%)	28/213 (13.2%)	16/116 (13.8%)	11/91 (12.1%)	25/248 (10.1%)	12/171 (7.0%)	7/123 (5.7%)	4/73 (5.5%)	1.87 (0.73–4.79)	1.77	0.1947	1.95	0.5826
DECA Self-Regulation	38/240 (15.8%)	25/216 (11.6%)	12/115 (10.4%)	8/93 (8.6%)	31/241 (12.9%)	16/175 (9.1%)	5/121 (4.1%)	4/73 (5.5%)	1.34 (0.55–3.28)	6.71	0.516	3.39	0.3359
DECA Total Protective Factors	34/222 (15.3%)	22/200 (11.0%)	13/113 (11.5%)	10/91 (11.0%)	28/230 (12.2%)	10/154 (6.5%)	8/117 (6.8%)	3/69 (4.3%)	1.40 (0.54–3.63)	1.26	0.492	2.81	0.4212
SDQ Externalizing Problems	74/230 (32.2%)	71/216 (32.9%)	38/113 (33.6%)	10/98 (10.2%)	67/266 (25.2%)	34/186 (18.3%)	25/127 (19.7%)	8/89 (9.0%)	1.05 (0.54–2.02)	6.81	0.8893	1.04	0.7924
SDQ Internalizing Problems	31/230 (13.5%)	28/216 (13.0%)	12/113 (10.6%)	10/98 (10.2%)	30/266 (11.3%)	19/186 (10.2%)	13/127 (10.2%)	8/89 (9.0%)	1.31 (0.64–2.69)	2.95	0.4557	0.1	0.9921
SDQ Total Problems	58/230 (25.2%)	48/216 (22.2%)	27/113 (23.9%)	18/98 (18.4%)	45/266 (16.9%)	29/186 (15.6%)	15/127 (11.8%)	9/89 (10.1%)	1.62 (0.82–3.20)	6.4	0.1638	4.48	0.2138

Note. JS+ = Jump Start + COVID Support; HC2 = Healthy Caregivers, Healthy Children (Active Control); DECA = Devereux Early Childhood Assessment; SDQ = Strengths and Difficulties Questionnaire. Concern/n = number of children with clinical concerns divided by the total sample for that time point. Tx = treatment.

## Data Availability

Requests for data can be sent to the corresponding author.

## Abbreviations

The following abbreviations are used in this manuscript:

DECA	Devereux Early Childhood Assessment
ECE	Early care and education
ECMHC	Early Childhood Mental Health Consultation
GEE	Generalized Estimating Equations
HC2	Healthy Caregivers—Healthy Children
IPCW	Inverse Probability of Censoring Weighting
IPTW	Inverse Probability of Treatment Weights
JS+CS	Jump Start Plus COVID Support
MHC	Mental health consultant
RA	Research assistant
SDQ	Strengths and Difficulties Questionnaire
SMD	Standardized Mean Difference
TPF	Total Protective Factors

## Appendix A

**Table A1:** Child Outcomes by Treatment Group and Time: Primary vs. Propensity-Score Adjusted Results.

Outcome	Primary GEE: JS+ vs. HC2 Diff (SE)	Primary Diff p	Primary Tx × Time *p*	PS-Adjusted: JS+ vs. HC2 Diff (95% CI)	PS-Adjusted Diff p	PS-Adjusted Tx × Time *p*
DECA Attachment	−0.26 (0.88)	0.766	0.329	−0.17 (−1.96, 1.62)	0.852	0.536
DECA Initiative	−1.18 (0.86)	0.171	**0.015**	−1.21 (−2.96, 0.54)	0.174	**0.006**
DECA Self-Regulation	−2.21 (0.79)	**0.005**	**0.027**	−2.18 (−3.81, −0.55)	**0.009**	**0.017**
DECA Total Protective	−1.01 (0.87)	0.246	0.101	−0.99 (−2.82, 0.84)	0.291	0.051
SDQ Externalizing	+0.76 (0.28)	**0.007**	0.897	+0.84 (0.26, 1.42)	**0.005**	0.867
SDQ Internalizing	+0.31 (0.18)	0.083	0.925	+0.35 (−0.01, 0.71)	0.055	0.975
SDQ Total Problems	+1.01 (0.39)	**0.009**	0.937	+1.12 (0.31, 1.93)	**0.007**	0.973

Note. Bold values indicate statistically significant results (*p* < 0.05).

**Table A2:** Logistic Regression Predicting Study Completion at T4.

Predictor	OR (95% CI)	*p*-Value
Treatment group (JS+ vs. HC2)	1.48 (0.89, 2.45)	0.13
Child age (years)	0.50 (0.39, 0.64)	<0.001
Gender	—	0.22
Race	—	0.31
Ethnicity	—	0.16
Primary language	—	0.18
Baseline DECA Total Protective Factors	1.01 (0.98, 1.04)	0.45
Baseline SDQ Total Problems	0.95 (0.90, 1.01)	0.11

Note. OR > 1 = higher likelihood of completing T4; OR < 1 = higher likelihood of dropout.
